# Regulation of Apoptosis in African Swine Fever Virus–Infected Macrophages

**DOI:** 10.1100/tsw.2002.214

**Published:** 2002-05-03

**Authors:** Laslo Zsak, John G. Neilan

**Affiliations:** Plum Island Animal Disease Center, USDA, ARS, P.O.Box 848, Greenport, NY 11944, USA

**Keywords:** African swine fever, virus, gene, protein, macrophage, cell death, apoptosis, virulence, host range, pathogenesis, immunology

## Abstract

A number of viruses have evolved antiapoptotic mechanisms to promote infected-cell survival, either to ensure efficient productive viral replication or to promote long-term survival of virus-infected cells. Recent studies identified critical African swine fever virus genes involved in the complex regulation of ASFV-host interactions. Here we review the present knowledge of the recently identified ASFV genes with special attention to those which affect viral virulence, host range, and pathogenesis by regulating viral-induced apoptotic mechanisms.

## INTRODUCTION

African swine fever (ASF) is a highly lethal, hemorrhagic disease of domestic pigs for which there is no vaccine or disease control other than animal slaughter and area quarantine. African swine fever virus (ASFV), the causative agent of ASF, is a large, enveloped, double-stranded DNA virus which is the sole member of the newly named *Asfarviridae* family[1]. Although the icosahedral morphology of the ASFV virion resembles those of iridoviruses, both the genomic organization, which includes terminal cross-links and inverted terminal repeats, and its cytoplasmic replication strategy suggest some relationship with the *Poxviridae* family[2,3,4]. ASFV is the only known DNA arbovirus[5]. Perpetuation and transmission of this virus in nature involves the cycling of virus between two highly adapted hosts, *Ornithodoros* ticks and wild pig populations (warthogs and bushpigs) in sub-Saharan Africa[6]. In enzootic areas, ASFV is maintained in a sylvatic cycle between warthogs, bushpigs, and *O. porcinus porcinus* ticks. In the warthog host, ASFV infection is subclinical, characterized by low viremia titers[7] and reduced lymphocyte apoptosis when compared to domestic swine[8,9].

In domestic pigs, ASF occurs in several disease forms, ranging from highly lethal to subclinical infections, depending on contributing viral and host factors[10]. ASFV infects cells of the mononuclear-phagocytic system, including fixed tissue macrophages, and specific lineages of reticular cells in spleen, lymph node, lung, kidney, and liver[10]. This ability to replicate and induce marked cytopathology in these cell types *in vivo* appears to be critical for ASFV virulence. Recent studies show that pathogenic ASFV isolates can also infect primary cultures of porcine endothelial cells *in vitro*, suggesting that vascular endothelial cells may play important role in the pathogenesis of the disease *in vivo*[11].Viral and host factors responsible for the differing outcomes of infection with highly virulent strains and strains of lesser virulence are largely unknown.

ASFV is genetically complex. Its genome of 170 to 190 kbp encodes 160 or more open reading frames (ORFs), and approximately 100 proteins have been observed in virus-infected cells[5]. Sequencing and genome analysis of the complete viral genome have led to the prediction and/or identification of genes encoding virion structural proteins and other proteins with functions involving viral virulence and/or host range, RNA transcription and modification, nucleotide metabolism, DNA replication, and protein processing[12,13, Z. Lu et al., unpublished data).

In this review, we give emphasis to the recently identified ASFV genes (see Table 1) involved in critical virus-cell interactions, specifically in the regulation of apoptosis in virus-infected target cells.

## ASFV GENES AFFECTING VIRULENCE, PATHOGENESIS, AND IMMUNE RESPONSE

Early interactions between the virus and specific cellular receptors appear to be essential for a productive infection. Specific binding and internalization of ASFV to macrophages are mediated by structural viral proteins p30, p54, and p12[15,18]. While virus-specific cell receptors are necessary to confer cell susceptibility to ASFV, they are not sufficient in subsequent efficient replication, suggesting that additional virus-host interactions are significant for host range specificity[38].

Several ASFV genes are associated with pathogenesis and virulence in swine. An ASFV gene, NL, with similarity to the neurovirulence gene *ICP34.5* of herpes simplex virus[39], and another terminal variable region gene, UK, were identified with functions involving virulence and host range in the pathogenic European isolate, E70[19,20]. Deletion of either NL or UK from pathogenic ASFV isolate does not affect viral growth in swine macrophage cell cultures, but markedly reduces virulence of the virus in swine. While these genes are important for ASFV virulence, they alone are not sufficient, indicating that other viral determinants must play significant roles in determining host range and viral virulence[19,20,40].

ASFV, like other large DNA viruses such as pox-, herpes-, and adenoviruses, encode nonessential, virulence-associated genes which play significant roles in downregulation of host cytokine-modulated immunological and inflammatory responses[41]. Downregulation of proinflammatory cytokine gene expression (TNF-α and INF-α) has been observed in mitogen or phorbol myristic acid-activated porcine macrophages within 4 h of ASFV infection[27]. Expression of these cytokines is in part regulated by the transcription factor, nuclear factor kappa beta (NFκB) protein complex. An African swine fever virus gene with similarity to the inhibitor of NFκB (IκB), has been identified in the pathogenic African isolate Malawi Lil-20/1 (5EL) and a cell-culture-adapted European virus BA71V (A238L)[13,26]. Expression of A238L in pig kidney cells (PK15) inhibited both the expression of an NFκB reporter gene and NFκB binding to DNA, indicating that A238L had functional IκB activity[27]. Since the NFκB protein complex functions as a pleiotrophic transcription factor that modulates a variety of genes in cells of the immune system (T cells, B cells, and monocyte/macrophages), a viral-encoded IκB could conceivably

**TABLE 1**
**African Swine Fever Virus Genes with Predicted and/or Identified Functions in Critical Virus-Cell Interactions**

| ASFV Genes | ORF | Characteristics/ Homology | Function | References |
|---|---|---|---|---|
| Structural proteins | p30 | early; membrane | Attachment; VN | 14,15 |
| | p54 | late; envelope | Internalization; VN | 15 |
| | p72 | late; envelope | VN | 16,17 |
| | p12 | early; envelope | Attachment | 18 |
| Virulence/host range-associated | NL | Early; HSV ICP 34.5 | Pig virulence | 19 |
| | UK | Early; no homolog | Pig virulence | 20 |
| | 9GL | Late; yeast ERV1 | Pig virulence | 21 |
| | TK | Early; thymidine kinase | Growth in macrophage; pig virulence | 22 |
| | MGF360 | Early; no homolog | Macrophage host range; pig virulence | 23,24 |
| | MGF530 | Early; no homolog | Macrophage host range; pig virulence | 23,24 |
| Immune response modulation | 5EL | Early; IκB | Inhibition of proinflammatory cytokines | 25,26,27 |
| | 8-DR | Late; T-lymphocyte CD2 | Cell-adhesion; T-cell response | 28,29,30 |
| | 8CR | Late; C-type lectins | Cell-adhesion; signal transduction; | 13,31 |
| Apoptosis regulation | 5HL | Early to late; Bcl-2 | Inhibition | 32,33 |
| | 4CL | Late; IAP | Inhibition / caspase-3 binding | 34,35,36 |
| | 5EL | Early; IκB | Inhibition / NFκB, NFAT | 26,37 |
| | MGF360 | Early; no homolog | Inhibition | 24 |
| | MGF530 | Early; no homolog | Inhibition | 24 |

play a highly significant role in counteracting an antiviral response in the ASFV-infected swine host. Indeed, data show that 5EL interacts with the RelA subunit of NFκB, where it is able to prevent binding of NFκB to target sequences and inhibit NFκB-dependent gene expression[27,42,43]. 5EL also provides a mechanism that enables ASFV to evade host defense systems by preventing the transcription of cellular NFAT transcription factor[25,44]. Given the central role of NFκB and NFAT in regulating the rapid expression of many genes encoding immunomodulatory proteins in monocytes and macrophages following activating stimuli, it is surprising that deletion of 5EL from the Malawi Lil-20/1 genome had no effect on disease onset, disease course, or viral virulence[26].

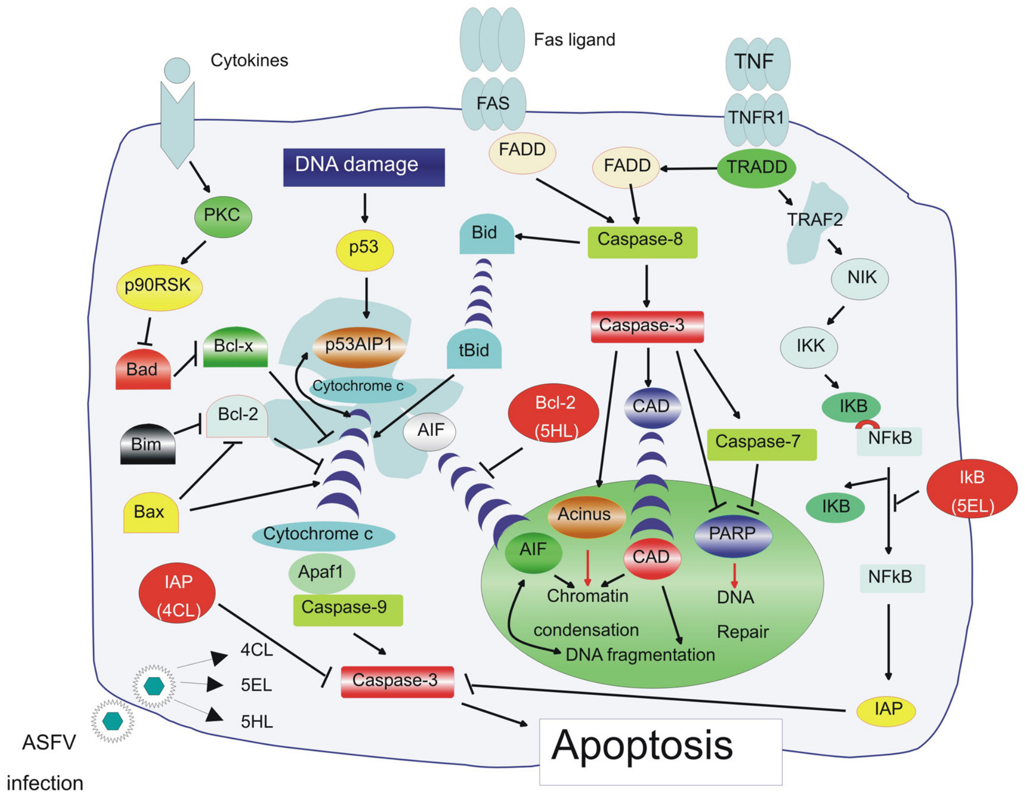

**FIGURE 1.** Viral-induced apoptosis in African swine fever virus infected macrophages.

## VIRAL-INDUCED APOPTOSIS IN ASFV-INFECTED MACROPHAGES

Apoptosis is a common pathway of virus-induced cell death. A number of viruses have evolved antiapoptotic mechanisms to promote infected-cell survival, either to ensure efficient productive viral replication or to promote long-term survival of virus-infected cells (for a review, see 45).

Sequencing and genome analysis of complete ASFV genomes have led to the identification of several genes that are likely involved in the complex regulation of viral-induced apoptotic mechanisms in infected swine macrophages. Earlier, we described an ASFV gene, 5-HL, with a high degree of similarity to the proto-oncogene Bcl-2[33](Fig. 1). The gene encodes a protein of 18 kDa (p21) that is present in infected porcine macrophages at both early and late times postinfection. The similarity of 5-HL to Bcl-2 suggested a role for it in cell maintenance during productive or persistent viral infection. Subsequent studies showed that 5-HL is a true functional viral member of the Bcl-2 gene family. The Bcl-2 family includes genes encoding proteins that can either inhibit apoptosis (Bcl-2, Bcl-$x_L$) or promote programmed cell death (Bax, Bcl-$x_S$, Bak). The prototype of the family, the Bcl-2 gene, was discovered in 1985 by genetic analysis of a chromosomal translocation that is characteristic of human follicular lymphoma[46]. Many more members of the still growing family have been identified. The ASFV 5-HL protein product, p21, suppressed apoptotic cell death in the mammalian lymphoid cell line FL5.12[32], in HeLa and BSC-40 cells[47], in the human myeloid leukemia cell line K562[48], and in insect cells[49]. In addition to ASFV, viral members of the Bcl-2 gene family in gammaherpesviruses and adenoviruses have been described[50,51]. The E1B 19-kDa protein of adenovirus has been shown to prolong infected-cell survival by inhibiting apoptosis induced by adenovirus E1A protein, tumor necrosis factor alpha, and Fas antigen[52]. Similarly, ASFV 5-HL may suppress apoptosis in ASFV-infected cells, thus promoting the survival of host cells during productive and/or persistent infection of either the pig or tick host. Certainly, this gene is highly conserved and present in even highly cell-culture-adapted viruses[32]; unsuccessful attempts to delete it from the viral genomes of pathogenic isolates suggest that it is essential for replication in swine macrophages (J. G. Neilan, unpublished data). ASFV infection induces apoptosis in primary swine macrophages *in vitro* as early as 20 h postinfection, a time at which viral replication has already occurred in these cells[35]. Thus, it is possible that p21, which is expressed throughout the infection cycle, including early time points, transiently modulates infected-macrophage survival, allowing productive viral replication to occur. Cytopathological changes consistent with apoptotic cell death, including karyorrhexis and chromatin condensation, have been observed in mononuclear cells (lymphocytes and monocytes/macrophages) in tissues of pigs infected with highly virulent ASFV isolates[8,53,54]. Therefore, suppression of apoptosis may be of significance to aspects of viral pathogenesis, and virulence and p21 may mediate this effect. Additionally, because ASFV-swine monocyte/macrophage interactions result in either lytic or latent infection[55], p21 could conceivably have a role in promoting the survival of latently infected mononuclear cells.

An ORF in the ASFV genome similar to inhibitor of apoptosis genes (iap) has been identified in a pathogenic African isolate, Malawi Lil-20/1[35], and an avirulent cell-culture-adapted European virus, BA71V[13]. The Malawi gene, 4CL shares 91.5% amino acid identity with the homologous gene A224L present in BA71V. Both 4Cl and A224L contain a single baculovirus IAP repeat motif (BIR motif). A224L has been shown to encode a 27-kDa late, structural protein[34]. Inhibitor of apoptosis genes were first described in baculoviruses, where they were shown to inhibit apoptosis of virus-infected cells[56]; later, human iap genes were identified and shown to inhibit apoptosis under *in vitro* assay conditions[57]. These genes encode proteins that contain one to three copies of IAP repeat motifs referred to as BIR motifs. The BIR motifs are involved in all known interactions between IAPs and other proteins, including binding to at least two members of the caspase family of cell death proteases, caspase-3 and caspase-7. Human IAPs have been shown to prevent the proteolytic processing of procaspase-3, -6, and -7 by blocking the cytochrome c-induced activation of procaspase-9[58]. These IAP family proteins can also suppress apoptotic pathways by inhibiting active caspase-3 directly[59]. Recently, Nogal et al.[36] reported that ASFV A224L modulates the proteolytic processing of caspase-3 and the apoptosis which are induced in infected VERO cell cultures. Their findings showed that A224L interacts with the proteolytic fragment of caspase-3 and inhibits the activity of this protease during viral infection. These data indicated a common mechanism of action for ASFV IAP and other IAP family members in inhibition of apoptotis. However, other studies revealed that deletion of 4CL from pathogenic ASFV isolate did not affect viral growth and apoptosis in macrophages or virulence in swine[35]. Therefore, the antiapoptotic function of this viral gene could be cell-type specific, or in the critical target cells other viral or cellular genes might complement its effect. Since iap genes have also been identified in the iridoviridae virus family[60], it is tempting to speculate that 4CL may be a host range gene involved in some aspect of infection in the arthropod host, *O. porcinus*.

In the acute stage, ASF is characterized by disseminated intravascular coagulation with multiple hemorrhages in all tissues and marked lymphopenia[61]. In experimentally infected pigs, Oura et al.[9] found that ASFV was predominantly localized in cells of the mononuclear phagocytic system and was not observed in endothelial cells in lymphoid tissue. Severe tissue destruction and cell depletion were seen in lymphoid tissues; the abundant lymphocyte death was caused by apoptosis. Although the mechanism of lymphocyte apoptosis induced by ASFV-infected macrophages was not defined, it is possible that release of TNF-$\alpha$ may contribute to the effect. In pigs infected with pathogenic European isolate E-75, enhanced TNF-$\alpha$ expression was detected which corresponded to the onset of clinical symptoms[37]. In addition, TNF-$\alpha$ containing supernatants from ASFV-infected macrophage cultures induced apoptosis in uninfected lymphocytes. These results suggested a relevant role for this cytokine in the pathogenesis of ASF. TNF is a type II membrane protein. Proteolysis of membrane-associated TNF, which is mediated by a membrane metallo-proteinase, produces a soluble form of this protein. Binding of this TNF to TNF-R leads to oligomerization of the receptor. Subsequently, the oligomerized TNF-R transmits signals to the cell that can result in the activation of NFκB and induction of apoptosis. Although ASFV does encode an IκB homolog gene, 5EL, which might be involved in the regulation of virus-induced TNF-α production, available data do not support directly this hypothesis. Deletion of 5EL from the pathogenic Malawi Lil-20/1 isolate did not alter the growth characteristics of the virus in swine macrophage cell cultures and virulence in pigs[26], suggesting that the gene has little or no effect on the regulation of apoptotic mechanisms in infected cells.

Variations in genome size and restriction fragment patterns are observed among different ASFV isolates. Like poxviruses, the diversity within the ASFV genome is localized primarily to the terminal genomic regions[62,63,64]. ASFV variable regions comprise the left 35-kb and the right 15-kb ends of the genome, and contain at least five multigene families (MGFs): MGF100, MGF110, MGF300, MGF360, and MGF530[65,66,67,68,69]. Variations in these regions, including gene deletion events, are observed during ASFV adaptation to monkey cell lines[62,70]. Recently, MGF360 and MGF530 genes have been shown to have a significant role in ASFV macrophage host range; these genes function in promoting infected cell survival[24].

MGF360 and MGF530 genes do not show similarity to other genes or motifs in the current databases. Individual MGF genes are conserved among ASFV isolates (80 to 98% amino acid similarity) (G. F. Kutish, personal communication). Transcriptional analysis of MGF360 and MGF530 genes showed that these ORFs are transcribed early during ASFV infection[69,71, JG. Neilan, unpublished data], but translated proteins have not yet been identified or characterized. Alignments of predicted amino acid sequences of all MGF360 and MGF530 family members show three conserved regions at the amino terminus of these proteins[69]. These conserved regions may indicate common ancestral relationships among the MGF genes or roles for these genes in common or related pathways. The function of different multigene families in ASFV infection is unclear at present.

In our studies, removal of individual MGF530 genes or groups of MGF360 genes from the left variable region of a highly virulent ASFV isolate, Pr4, did not alter the viral growth in macrophage cell cultures. However, deletion of six MGF360 and two MGF530 ORFs from the left end of the Pr4 genome markedly reduced viral growth in primary macrophage cell cultures (Fig. 2); removal of two additional MGF360 genes resulted in no growth at all[24]. Cell viability studies and apoptosis assays revealed a significant reduction in survival time, and accelerated and increased apoptotic cell death in gene deletion mutant, Δ35-infected macrophage cell cultures (Fig. 2). Although little is known about MGF gene function during virus replication, it is tempting to speculate that gene dosage is a factor in replication in macrophages. Alternatively, specific MGF members may function and/or interact with each other in a yet-to-be-identified pathway(s). Given that macrophages are the primary target cells of ASFV in swine, it is likely that these genes are also of significance for viral pathogenesis and virulence in domestic swine. Most recently Neilan et al. reported that ASFV MGF360 and MGF530 genes as novel swine virulence determinants play important role in pig virulence[23]. Although swine virulence determinants and macrophage host range determinants differ phenotypically, they share four MGF genes, suggesting that they may have related functions in maintaining infected-cell survival in specific ASFV target cells, which are critical for a virulence phenotype in domestic pigs. These data indicate that ASFV MGF360 and MGF530 genes perform an essential macrophage host range function(s) that involves the promotion of infected cell survival. Reduced viral growth in macrophages together with increased early apoptotic death of virus-infected cells likely account for the attenuation of MGF gene deletion mutants in swine.

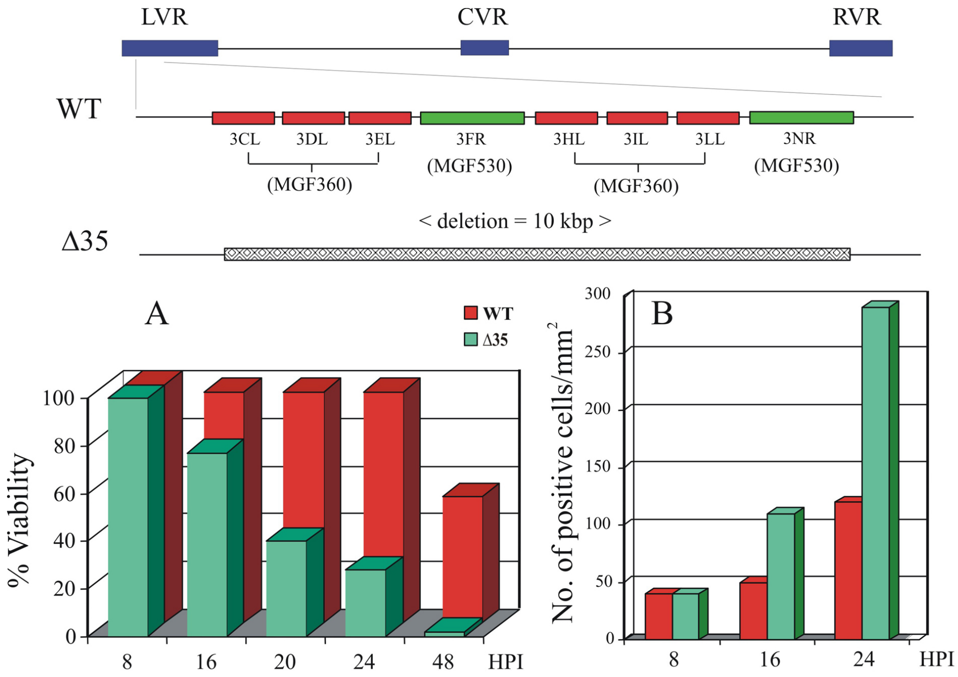

**FIGURE 2**. Cell viability (A) and TUNEL assays (B) in MGF360/530 gene deletion mutant, Δ35-infected porcine macrophage cell cultures. Cultures were infected (MOI = 5) with wild-type (WT) or Δ35 viruses, and examined by trypan blue exclusion viability assay and TUNEL assay at various times postinfection. Data indicate a significant reduction in survival time, and accelerated and increased apoptotic cell death in gene deletion mutant, Δ35-infected macrophages (Zsak et al., unpublished data).

## CONCLUSIONS

Identification of critical virulence and host range genes of African swine fever virus revealed the complexity of virus-induced mechanisms in infected host. Although how these mostly novel viral genes mediate virulence, host range, and pathogenesis is not fully understood, it is likely that many of them perform essential function(s) during crucial ASFV-host interactions. Further identification and characterization of virulence and host range genes, and the mechanisms of their action, will permit engineering of viral strains attenuated for virulence and/or restricted host range as possible vaccine candidates.

## ACKNOWLEDGMENTS

We thank Dr. D.L. Rock for his encouragement during the preparation of this manuscript.
